# Hafnium (IV) oxide obtained by atomic layer deposition (ALD) technology promotes early osteogenesis via activation of Runx2-OPN-mir21A axis while inhibits osteoclasts activity

**DOI:** 10.1186/s12951-020-00692-5

**Published:** 2020-09-15

**Authors:** A. Seweryn, M. Alicka, A. Fal, K. Kornicka-Garbowska, K. Lawniczak-Jablonska, M. Ozga, P. Kuzmiuk, M. Godlewski, K. Marycz

**Affiliations:** 1 Institute of Physics, Polish Academy of Sciences, 02668 Warsaw, Poland; 2 Department of Experimental Biology, Faculty of Biology and Animal Science, Wrocław University of Environmental and Life Sciences, Norwida 27B, 50-375 Wrocław, Poland; 3 Cardinal Stefan Wyszynski University, Collegium Medicum, 01938 Warsaw, Poland; 4 International Institute of Translational Medicine, Jesionowa 11, Malin, Wisznia Mała, 55-114 Wrocław, Poland

**Keywords:** Hafnium (IV) oxide, Atomic layer deposition, Osteoblasts, Osteoclasts, Biomaterials, Osteoporosis

## Abstract

**Background:**

Due to increasing aging of population prevalence of age-related disorders including osteoporosis is rapidly growing. Due to health and economic impact of the disease, there is an urgent need to develop techniques supporting bone metabolism and bone regeneration after fracture. Due to imbalance between bone forming and bone resorbing cells, the healing process of osteoporotic bone is problematic and prolonged. Thus searching for agents able to restore the homeostasis between these cells is strongly desirable.

**Results:**

In the present study, using ALD technology, we obtained homogeneous, amorphous layer of hafnium (IV) oxide (HfO_2_). Considering the specific growth rate (1.9Å/cycle) for the selected process at the temperature of 90 °C, we performed the 100 nm deposition process, which was confirmed by measuring film thickness using reflectometry. Then biological properties of the layer were investigated with pre-osteoblast (MC3T3), pre-osteoclasts (4B12) and macrophages (RAW 264.7) using immunofluorescence and RT-qPCR. We have shown, that HfO_2_ (i) enhance osteogenesis, (ii) reduce osteoclastogenesis (iii) do not elicit immune response and (iv) exert anti-inflammatory effects.

**Conclusion:**

HfO_2_ layer can be applied to cover the surface of metallic biomaterials in order to enhance the healing process of osteoporotic bone fracture. 
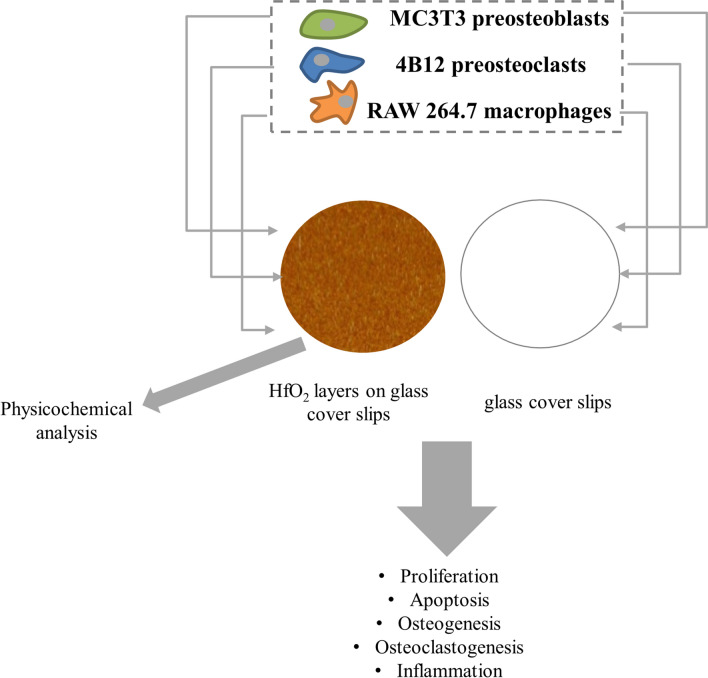

## Background

Regenerative medicine is a fast-growing field that is being successfully applied in traumatology or reconstructive surgery, where it is showing to be a promising avenue for the treatment of elderly patients [1]. Due to rapid aging of populations, there is an urgent need to develop personalized therapies for aged-related diseases. One of the most common disorder affecting elderly population worldwide is osteoporosis (OP) which deteriorates bone mass and architecture [2]. In accordance to recent data, around 200 million people suffer from OP and 8.9 million fractures are caused by the disease [3]. Besides being a great concern of the health care system, OP comes with economic burden. In the United States of America, costs of OP-related fractures is estimated to $13.8 billion. Therefore, OP represents not only clinical and public issue but due to significant morbidity, mortality and health care costs it represents a major challenge for world economies.

Metallic implants have been utilized for different medical purposes including orthopedics for short as well as long term fixations since many years [4]. So far, the most frequently applied metallic materials in traumatology are stainless steel (SS), titanium or cobalt alloys [5]. For the fixation of simple fractures, usually SS is applied due to much lower costs than titanium alloys. However, due to its corrosive nature and risk of allergic reaction due to released ions, SS is recommended for short term fixation procedures [6]. In turn, titanium alloys are characterized by good corrosion resistance and biocompatibility in contact with human body fluids, but their biomechanical properties are less attractive, when compared to SS. Metallic materials seem to be still an irreplaceable in reconstructive surgery, although there are many reports indicating on their disadvantages including postoperative complications, distortion of post-operative metallic screws and inflammatory reactions [5].

Methods have been devised to modify and improve the properties of the base material in order to enhance the medical outcome of the therapy. One of the approach to enhance the properties of metallic materials is their surface modifications by the application of various techniques including Atomic Layer Deposition (ALD). This technique allows to deposit a thin film onto various materials, such as metal, glass and polymers [7]. The ALD method is based on sequential introduction of selected chemical compounds (precursors) into the reaction chamber. As a result of chemical reactions between precursors on the substrate, a thin film grows closely attached to the substrate. The introduction of selected precursors is separated by the neutral gas fraction e.g. argon or nitrogen, which allows removal of the unreacted precursor molecules and reaction by-products from the reactor chamber [8]. Thickness of a newly formed layer is controlled by number of ALD cycles and the growth process is self-limiting. In optimal case only one monolayer may be grown during one ALD cycle (precursor doses separated by purging of a growth chamber). Such deposition model allows precise control of the layer thicknesses. The required thickness is controlled by estimated number of the ALD cycles [9]. One of the biggest advantages of the ALD technique is ability to obtain highly reproducible, homogenous coatings while the deposition process does not depend on source of the substrates [10]. What is more, the growth process can be performed even at room temperature [11], which allows to consider future functionalization of biological factors, even such as cells or drugs [12, 13]. Despite low temperature of growth, the resulting layers are of high quality and are highly homogeneous. Due to its multiple advantages, ALD technology allows to obtain nanolayers characterized by specific biological properties. In particular, the ALD method leads to deposit of transition metal oxides. Recently our group reported that zirconium (IV) oxide (ZrO_2_) thin films, deposited using ALD technology, improve metabolic as well as pro-osteogenic potential of bone precursor cell line through activation of miR-21 [14], which become an motivation for the present study.

Intelligent, smart scaffolds, including metallic materials, dedicated for osteoporotic bone regeneration, should be designed to represent not only great biocompatibility but also bioactivity in order to modulate microenvironment of surrounding tissue [15, 16]. It is strongly required for a scaffold to enhance osteogenesis via promotion of osteoblast differentiation, while silencing osteoclasts differentiation and maturation. What is more, in the course of bone fracture healing triggering of intrinsic, natural processes in order to reduce inflammation is strongly required. For that reason, we decided to investigate whether hafnium (IV) oxide (HfO_2_)layer can be utilized in the fabrication of personalized biomaterials for elderly patients during OP fractures. In presented paper, we analyzed physicochemical and biological properties of the layer revealing its pro-osteogenic properties. Obtained results shed a promising light for HfO_2_ future application in the field of nanometric coatings for biomedical applications.

## Results

### Physicochemical analysis

**Fig. 1:**
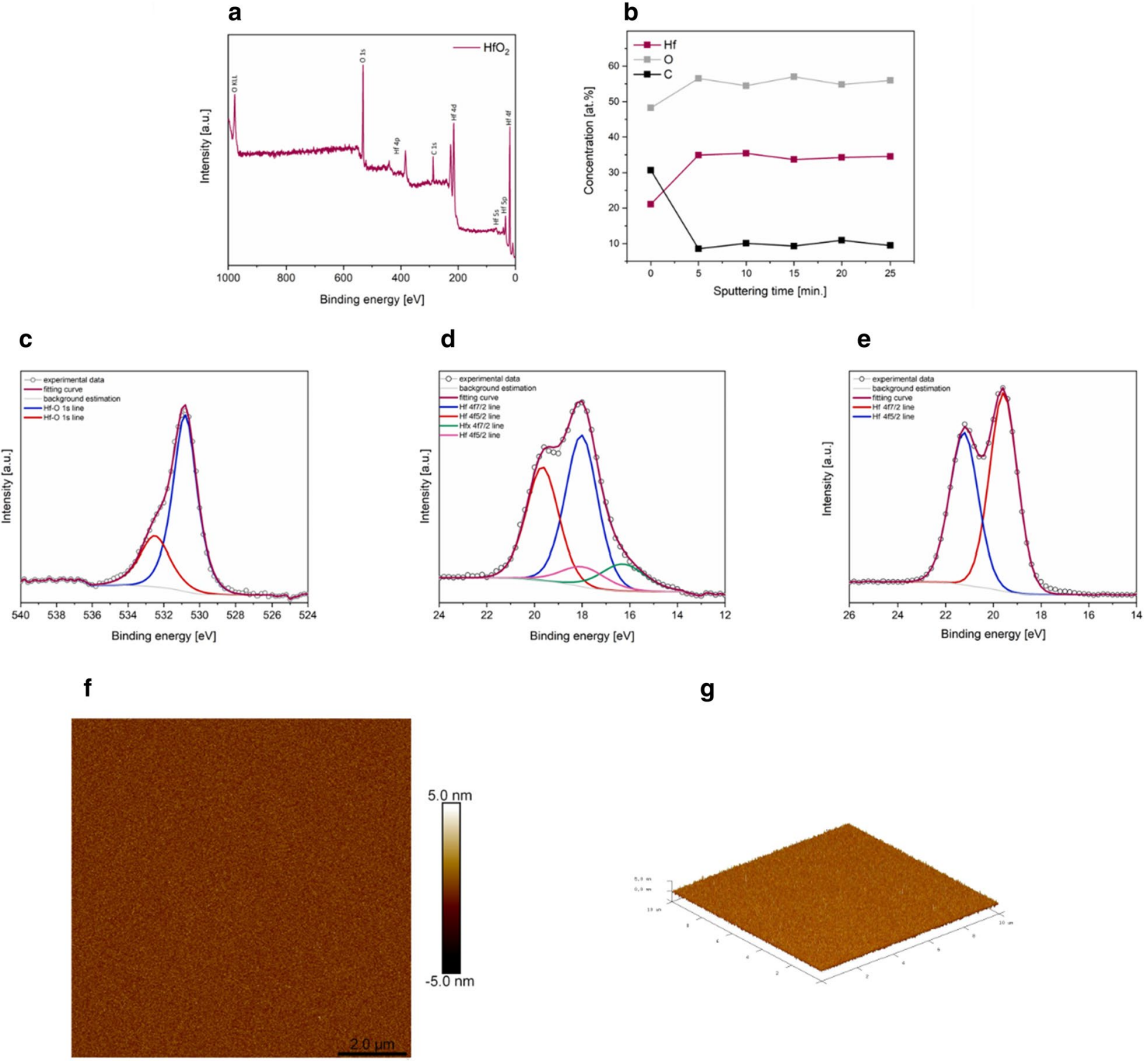
Spectrum of XPS measured over a wide range of elements binding energy (**a**), only O, Hf and C were detected. The distribution of elements: Hf, O and C as a function of Ar^+^ spattering time (**b**), high resolution O 1 s line decomposed into two components: bonded with Hf (blue line) and atmospheric contamination (red line) (**c**), hafnium 4f 7/2 (red line) and 4f 5/2 (blue line) spin –orbit doublet before sputtering (**d**) and after sputtering (**e**), the second spin–orbit doublet should be added to account for Hf bonded with less than 7 oxygen atoms. The AFM images of obtained HfO_2_ thin layer: top view of scan area equal to 4 μm^2^ (**f**) and three-dimensional view of scan area of 100 μm^2^(**g**)

The X-ray photoelectron spectroscopy (XPS) analysis (Fig. 1) indicated that HfO_2_ was formed on the surface during the ALD growth process. Only oxygen (O) and hafnium (Hf), in the ratio of 2.2, and carbon (C) were detected (Fig. 1a) [17]. The hhigh C content (about 31%) and oxygen surplus probably comes from atmospheric pollution. The oxygen line analysis (Fig. 1c) confirms that many of oxygen atoms (about 75%) were bound with hafnium atoms with the binding energy equal to 530.6 eV [18]. The 25% of the oxygen line is answering the surface contamination with the value of binding energy 532.6 eV.

The results of depth profiling are presented in Fig. 1b. The concentration of elements was measured after 5,10,15,20 and 25 min of sputtering. The Hf 4f lines (19.6 eV) (Fig. 1c) were analyzed to estimate the hafnium content. [18] Unlike the more surface sensitive Hf 4d lines (213.8 eV), the Hf 4f lines come from less strongly bound electron shells, therefore photoelectrons have higher kinetic energy and released from larger depths, which gives more accurate results of measurements minimizing disorder introduced by the sputtering. The final tests depth was estimated to be about 35–40 nm. Therefore, we can claim the homogeneity of the element’s distribution in the coating. After the first 5 min of sputtering, the carbon content dropped to 10 at % and remained constant in subsequent measurements. The ratio of oxygen atoms to hafnium atoms was also stable at the level 1.6. This change in respect to as received film results from the preferential sputtering of oxygen what is seen in the change of Hf 4f line during spattering (Fig. 1e). After 5 min sputtering the Hf 4f line bounded to 7 oxygen atoms (metal centres in HfO_2_ are coordinated by seven oxygen atoms [11]) simulated by the mixed Gaussian (40%) and Laurencin (60%) function is broadening from FWHM equal to 1.4 eV to 1.6 eV. Moreover, the second doublet (20% of the line area) should be added to account for the Hf atoms bounded to less than 7 oxygen atoms with FWHM equal to 2.2 eV, what evidences the disorder introduced during the sputtering [19].

The thickness of the oxide layer was determined by comparing the reflection spectrum of white light from the substrate (Si) to the reflection spectrum of light from a HfO_2_ layer deposited on the silicon substrate. A 100 nm thick HfO_2_ layer was successfully deposited onto the substrate. Additionally, lack of an X-ray diffraction (XRD) signal confirms the amorphous nature of the films. XRD can only occur on the ordered structure of the atoms.

The morphology was investigated using Atomic Force Microscope (AFM) (Fig. 1f, g). Surface images were made for areas of the size 10 μm × 10 μm (Fig. 1g), 2 μm × 2 μm (Fig. 1f) and 1 μm × 1 μm. The surface roughness of the layer was determined from the obtained AFM data. Ra is an arithmetic average of the absolute values of the surface height deviations and Rq is the root mean square (RMS) average of height values. The calculated surface roughness Ra is 0.422 nm, 0.537 nm and 0.536 nm while Rq is equal to 0.535 nm, 0.678 nm and 0.675 nm respectively for 100 μm^2^, 4 μm^2^ and 1 μm^2^ area. Uniformly distributed peaks can be observed on the surface of the layer there. Their maximal peak-to-valley value is only 6.5 nm over 100 μm^2^ area. Such a small maximum value of the height of irregularities occurring on the surface indicates uniform growth of the layer and lack of contamination on the surface.

### Hafnium (IV) oxide (HfO_2_) significantly affects proliferation activity and viability of osteoblast precursors and pre-osteoclasts

**Fig. 2:**
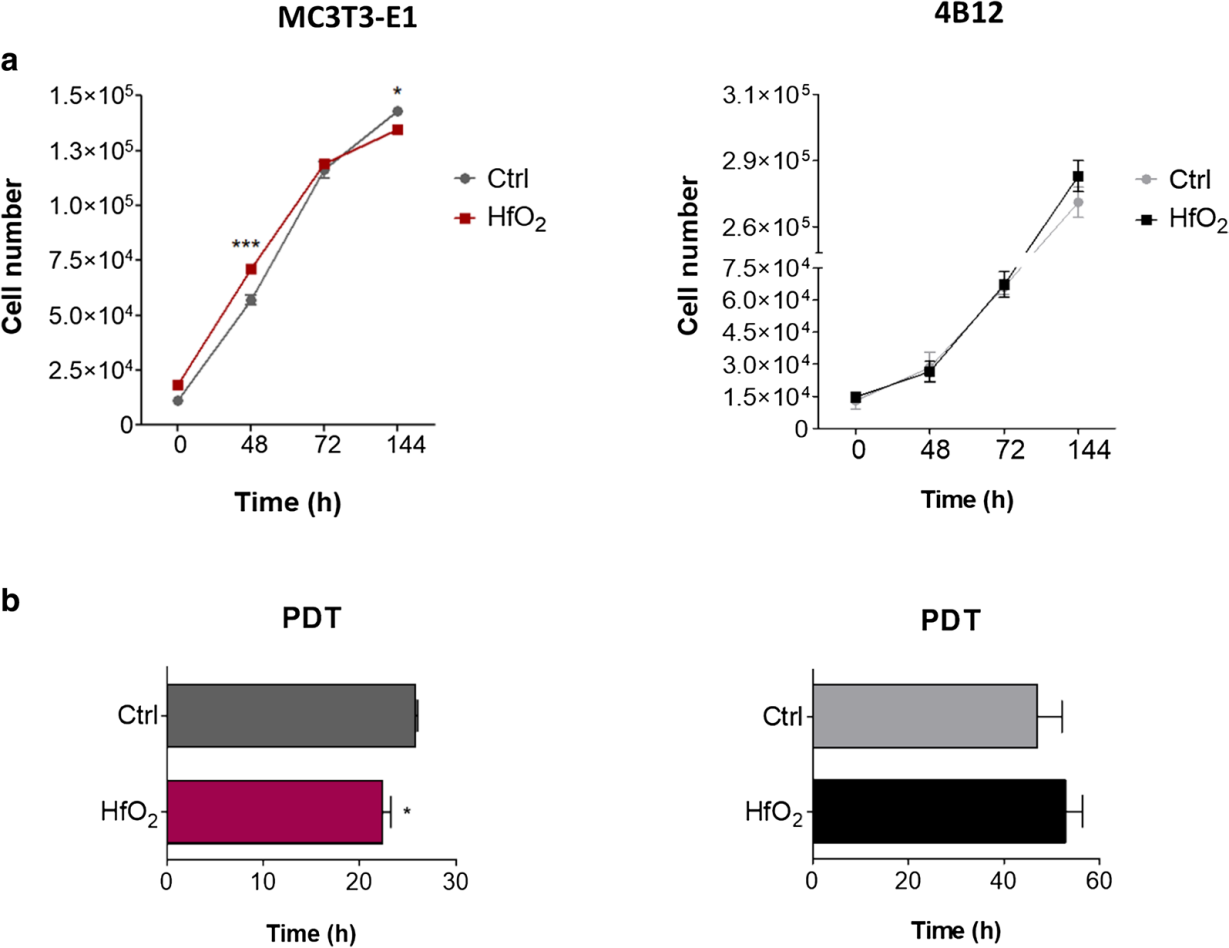
Temporal dynamic of MC3T3 and 4B12 proliferation activity. Cells were cultivated under normal growth condition or in the presence of HfO_2_. Proliferation rate of MC3T3 and 4B12 was estimated using resazurin-based in vitro toxicology (TOX8) assay kit (**a**). Population doubling time (PDT) value of MC3T3 and 4B12 cells was determined using TOX8 assay results (**b**). Error bars represent the means ± SD. *p < 0.05, ***p < 0.001; Student’s *t* test

The effect of HfO_2_ on proliferation activity pre-osteoblasts (MC3T3-E1) and pre-osteoclasts (4B12) was evaluated with resazurin based assay. Cell proliferation was monitored during 144 h of culture in normal condition or in the presence of HfO_2_. We noted that HfO_2_ significantly enhanced proliferation of MC3T3-E1 after 48 h (*p* < 0.001), while HfO_2_ did not affect proliferation of pre-osteoclast cell line (Fig. 2a). Based on data obtained from repeated resazurin-based in vitro toxicology assay (TOX8), population doubling time (PDT)- the time required for a cell population to double their number-was determined. The assay revealed that HfO_2_ significantly decreasedPDT value of pre-osteoblasts (*p* < 0.05), while did not change proliferation rate of 4B12 (Fig. 2b).

**Fig. 3:**
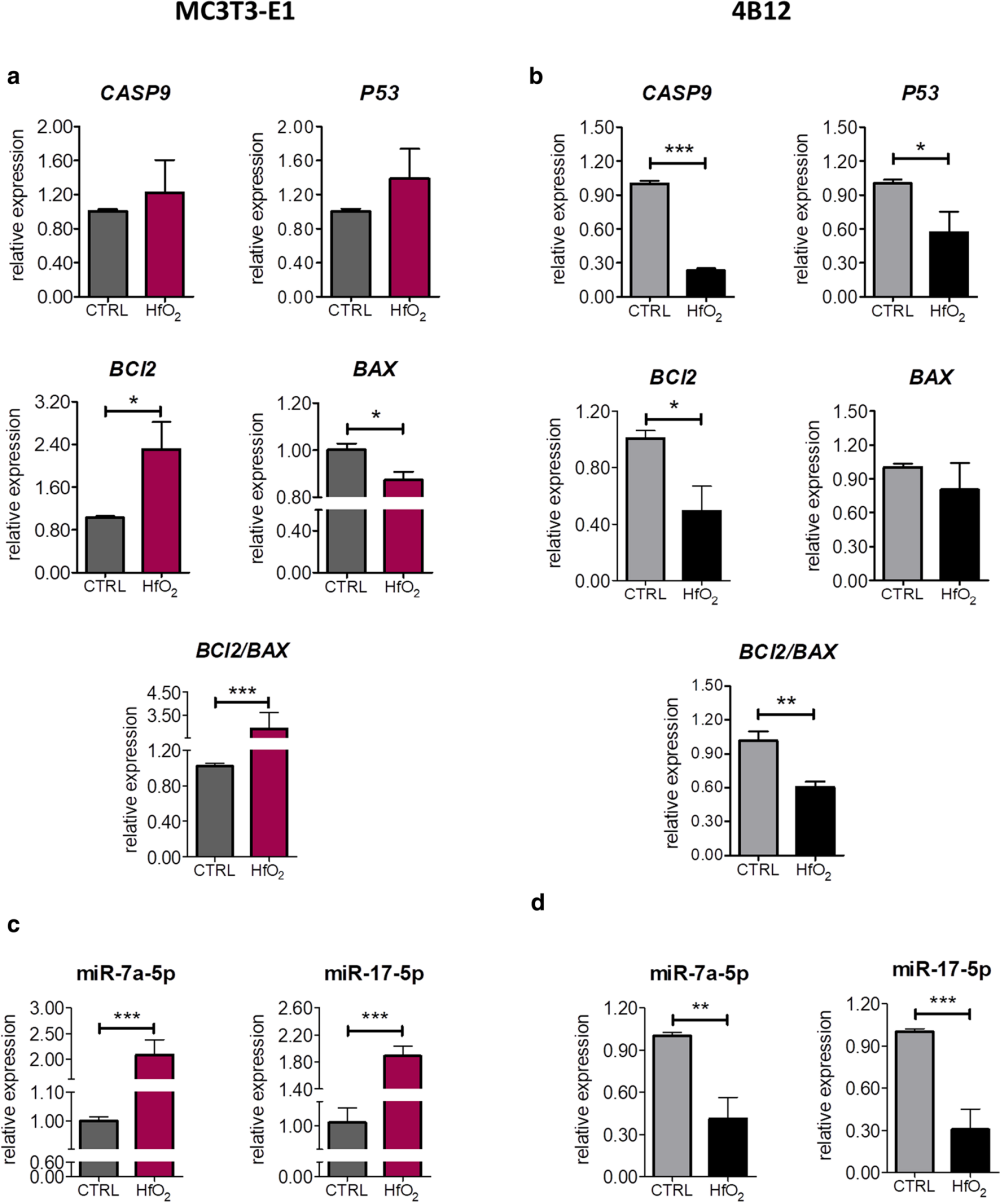
The effect of HfO_2_ on the expression of apoptosis-related genes and miRNAs in MC3T3-E1 and 4B12 cells after 144 h of culture. Expression pattern of *CASP9*, *P53*, *BCl2* and *BAX* was determined using RT-qPCR method (A-B). BCL2/BAX ratio was calculated based on relative expression of those genes. *GAPDH* was used as housekeeping gene. The relative level of miR-7a and miR-17-5p was estimated with Mir-X-system using RT-qPCR method (C-D). U6 was used as normalization control. Error bars represent the means ± SD. *p < 0.05, **p < 0.01, ***p < 0.001; Student’s *t* test

To determine whether HfO_2_ affects cell apoptosis, the cells were cultured for 144 h (6 days) in normal condition or in the presence of HfO_2_. Relative expression of apoptosis-related genes was estimated using RT-qPCR. We detected that several genes expression was affected by HfO_2_ in both MC3T3-E1 and 4B12 cell lines. Exposition to HfO_2_ significantly increased expression of anti-apoptotic *BCl2* (B cell lymphoma 2 gene) (*p*  <  0.05) and downregulated pro-apoptotic *BAX* (BCL2 associated X protein) (*p*  <  0.05) that resulted in higher *BCl2/BAX* ratio (*p*  <  0.001) in MC3T3-E1 exposed to HfO_2_ indicating lower apoptosis rate (Fig. 3a). On the contrary, treatment with HfO_2_ evidently decreased mRNA level of anti-apoptotic *BCl2* (*p*  <  0.05) caused low *BCl2/BAX* ratio (*p*  <  0.01). Interestingly, mRNA level of pro-apoptotic caspase 9 encoded by *CASP9* (*p*  <  0.001) and *P53* (tumor protein p53) (*p*  <  0.05) were significantly decreased in 4B12 after exposition to HfO_2_ compared to control group (Fig. 3b). Additionally, we noted that HfO_2_ markedly enhanced expression of miR-7a-5p (*p*  < 0.001) and miR-17-5p (*p* <  0.001) in MC3T3-E1, a widely known miRNA involved in promotion of cell proliferation and inhibition of apoptosis (Fig. 3c) [20, 21]. Conversely, those miRNAs are downregulated in 4B12 (Fig. 3d). To conclude, HfO_2_ upregulated BCL2/BAX ratio and expression of miR-7a-5p and miR-17-5p in MC3T3, while in 4B12 was observed inverse relation. In those cells, HfO_2_ diminished mRNA levels of *CASP9* and *P53,* thus suggesting that HfO_2_ may inhibit cells apoptosis affecting different signaling pathways depending on cell type.

### HfO_2_ modulates expression of osteogenic and osteoclastogenic markers

**Fig. 4:**
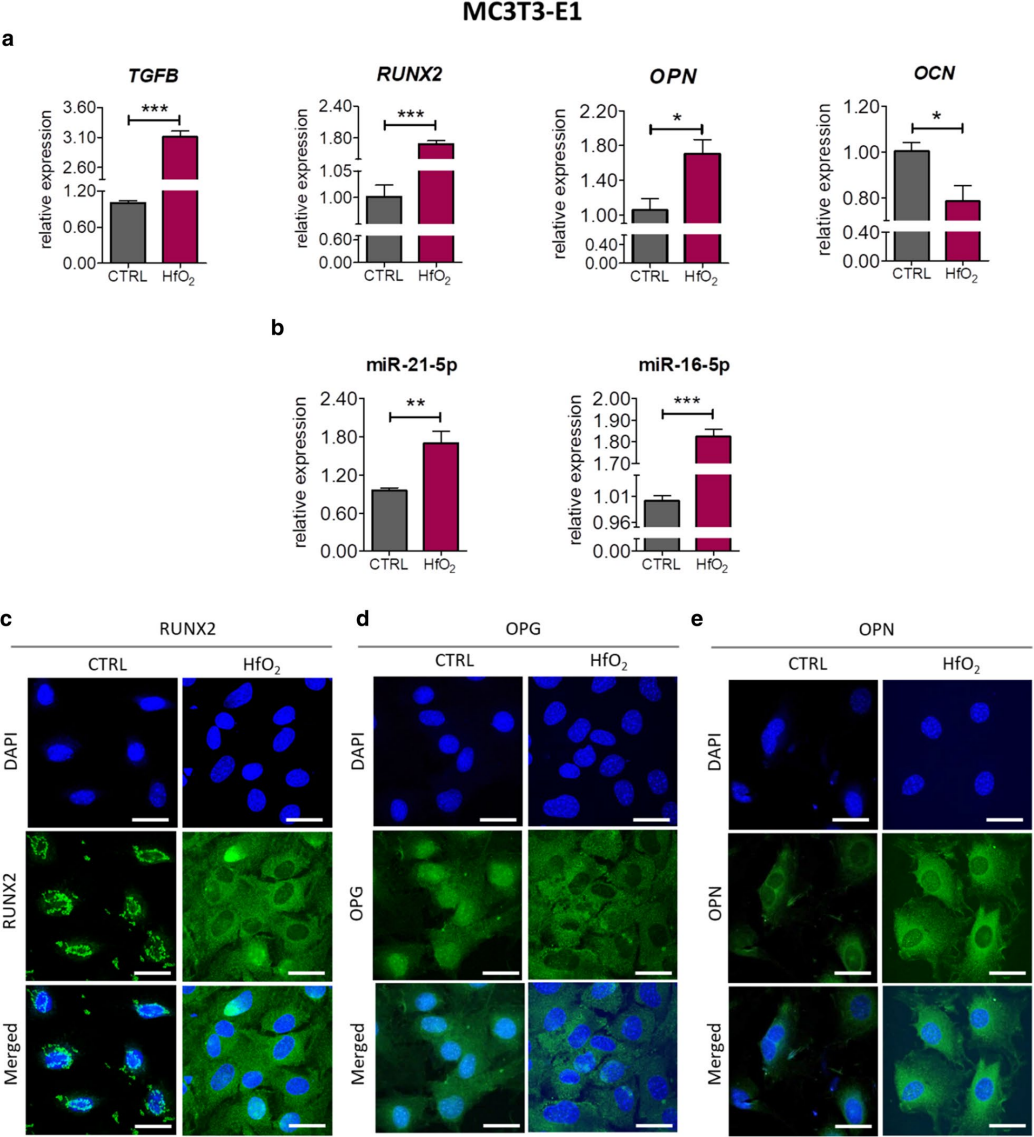
Expression of mRNA, miRNA and cellular localization of proteins involved in osteogenic differentiation in MC3T3 cells cultured on standard glass slides or on the glass slides covered with HfO_2_ for 144 h. Relative expression of *TGFB*, *RUNX2*, *OPN* and *OCN* was determined using RT-qPCR method (**a**). *GAPDH* was used as normalization control. Relative expression of osteogenesis-related miRNA (**b**). U6 was used as endogenous control for miRNA qPCR. Additionally, immunufluorescence for RUNX2 (**c**), OPN (**d**) and OPG (**e**) were performed. Error bars represent the means ± SD. *p < 0.05, **p < 0.01, ***p < 0.001; Student’s *t* test. Representative images of immunofluorescence staining for RUNX2, OPG and OPN (Z-projects) (green: Atto 488, blue: DAPI) (**c**). Scale bar is equal 50 µm

The effect of HfO_2_ on expression of specific markers involved in pre-osteoblast differentiation and bone formation after 144 h of culture was determined using RT-qPCR technique and immunofluorescence staining. We observed overexpression of runt-related transcription factor 2 (*RUNX2*) (*p*  <  0.001) and transforming growth factor β encoded by *TGFB* in MC3T3-E1 cells exposed to HfO_2_ (Fig. 4a). In addition, osteopontin (*OPN*) (*p*  <  0.05) was upregulated, while osteocalcin encoded by *OCN* (*p*  <  0.05) was downregulated in HfO_2_ group (Fig. 4a). Additionally, HfO_2_ positively regulated expression of two key miRNA involved in osteogenesis: miR-21-5p (*p* < 0.01) and miR-16-5p (*p* < 0.001) (Fig. 2b). miR-21-5p is widely known osteogenesis stimulator, whereas miR-16 suppress expression of key osteogenic markers and mineral calcium deposition by mesenchymal stem cells during osteogenic differentiation [22, 23]. Immunofluorescence staining revealed significant differences in distribution of RUNX2 and osteoprotegerin (OPG), as well as substantial alternation in the expression of OPN between experimental groups. In normal culture condition, RUNX2 exhibited perinuclear localization in MC3T3-E1, while HfO_2_ induced subcellular distribution of this protein (Fig. 4c). Similarly, exposition to HfO_2_ caused shift in fluorescence from nucleus area to cytoplasm of OPG (Fig. 4d). It is also worth noting that HfO_2_ increased expression of OPN protein (Fig. 4d).

**Fig. 5:**
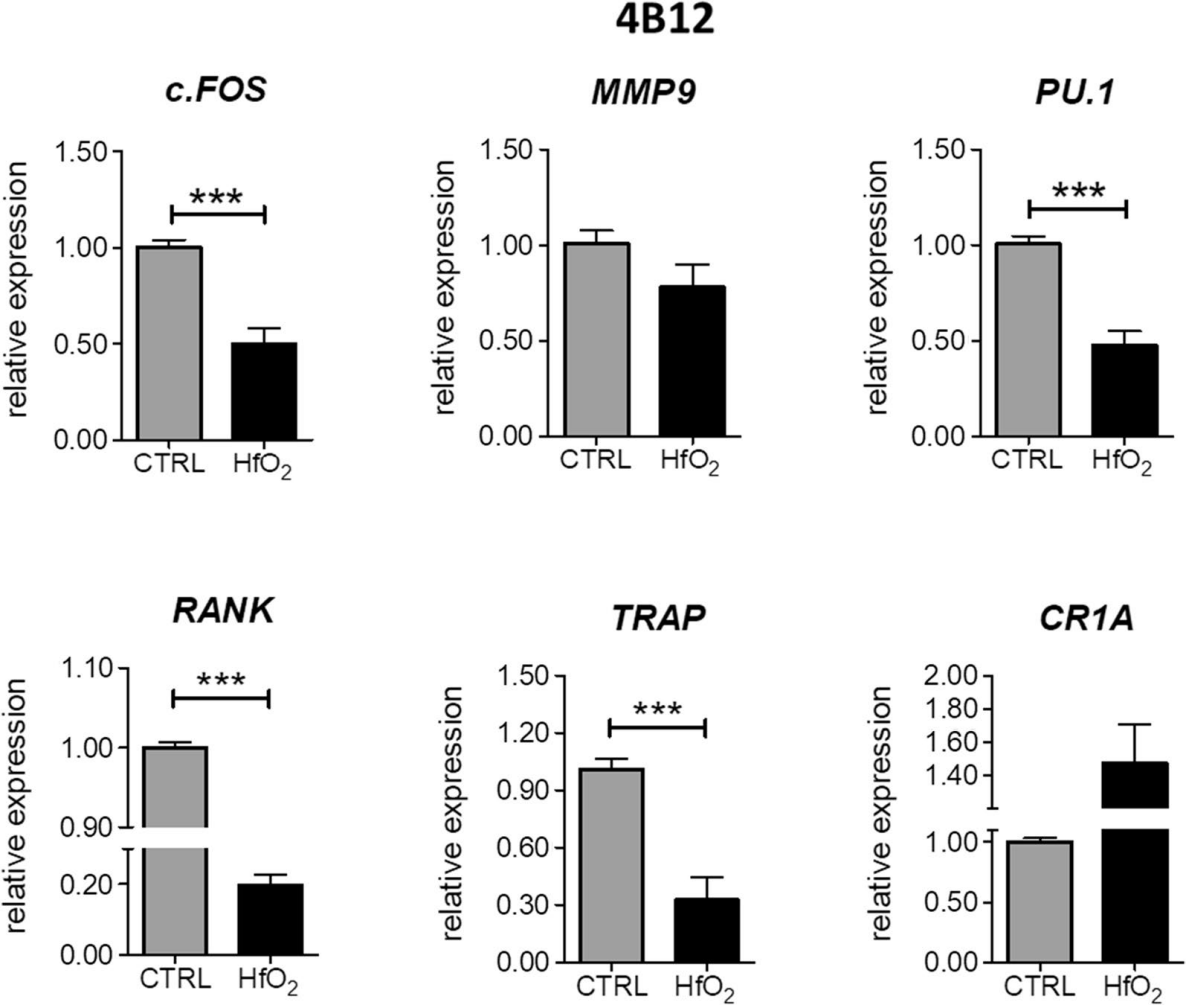
Expression of osteoclast-specific genes in 4B12 cells after 144 h of culture in normal condition or in the presence of HfO_2_. mRNA level of *c.FOS, MMP9, PU.1, RANK, TRAP* and *CR1A* was determined using RT-qPCR method. Gene expression data was normalized to the expression of *GAPDH*. Error bars represent the means ± SD. *p < 0.05, **p < 0.01, ***p < 0.001; Student’s *t* test

RT-qPCR results revealed that after 144 h of culture, HfO_2_ negatively regulated expression of genes involved in osteoclastogenesis. We observed significant decline in expression of *c.FOS* (*p*  <  0.001), *PU.1* (*p*  <  0.001), *RANK* (receptor activator of nuclear factor κ B) (*p*  <  0.001) and *TRAP* (tartrate-resistant acid phosphatase), whereas upregulation of calcitonin receptor isoform 1a encoded by *CR1A* (*p*  <  0.01) in HfO_2_ group (Fig. 5). These data indicated that HfO_2_ induces overexpression of osteoblastogenic markers, affects the content of OPN, as well as distribution profile of RUNX and OPG, while inhibits expression of genes crucial for pre-osteoclast differentiation.

### Biocompatibility analysis of HfO_2_ on RAW 264.7 cells

**Fig. 6:**
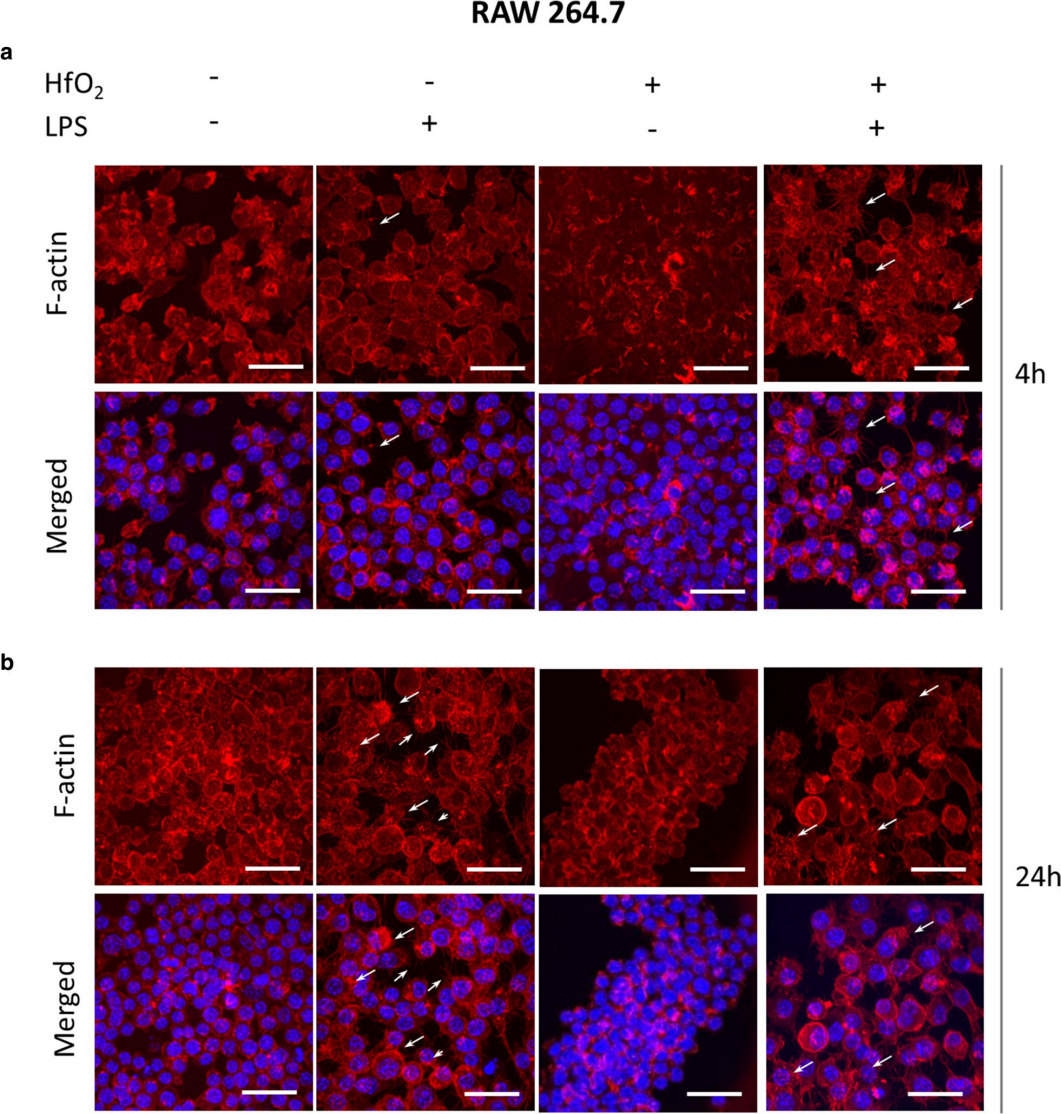
Representative images of staining for F-actin structures in RAW 264.7 after 4 h (**a**) and 24 h (**b**) incubation with LPS. F-actin was stained with Phalloidin 590, while cell nuclei were counterstained with DAPI (blue). White arrows indicated morphological alternations of macrophages after LPS treatment. Scale bar is equal 50 µm

Biocompatibility of HfO_2_ was evaluated using macrophage cell line RAW 264.7. Cells were seeded on standard glass slides or glass slides coated with HfO_2_ and allowed to attach overnight. Next, cells were exposed to lipopolysaccharide (LPS) for another 4 and 24 h. Cells treated with LPS (1 μg/mL) served as positive control for macrophage activation. F-actin staining revealed multiple morphological alternations in LPS positive groups after only a 4 h of treatment. When RAW 264.7 were exposed for 4 h, pseudopodia were formed and extended from few sides of cells (Fig. 6a). Pseudopodia were further extended after 24 h of LPS-stimulated macrophages (indicated with white arrows), whereas treatment with HfO_2_ diminished those LPS-induced morphological changes (Fig. 6b). Interestingly, we observed significant increase in cell size of macrophages induced with LPS after 24 h of exposition. In addition, co-treatment of HfO_2_ and LPS reversed LPS-induced alternations. Those cells displayed spherical shape and smooth surface similarly to negative control (RAW cultured in standard condition).

**Fig. 7:**
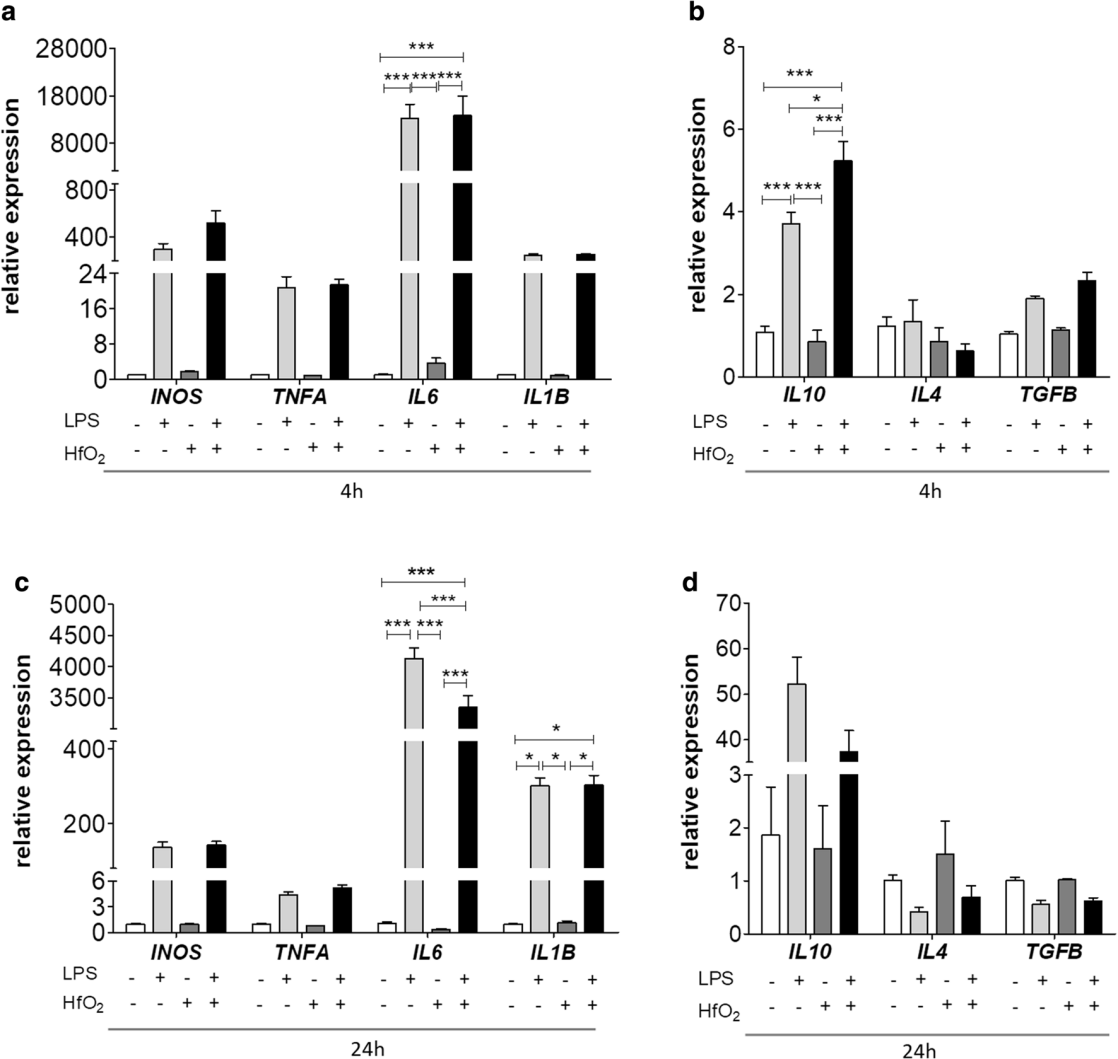
Changes in the expression of pro- and anti-inflammatory cytokines. Relative expression of cytokines was assessed after 4 h (**a**, **b**) and 24 h (**c**, **d**) stimulation with LPS using RT-qPCR method. *GAPDH* was used as housekeeping gene. Error bars represent the means ± SD. *p < 0.05, **p < 0.01, ***p < 0.001; Two-way ANOVA analysis of variance followed by Bonferroni post hoc testes

In the second step of the experiment polarization of macrophage M1/M2 using RT-qPCR method was investigated. We noted that LPS-treated macrophages exhibited overexpression of cytokines involved in M1 polarization, such as nitric oxide synthase (*INOS*), tumor necrosis factor alpha (*TNFA*), interleukin 6 (*IL6*) and interleukin (*IL1B*) after 4 h of LPS stimulation (Fig. 7a). Interestingly, HfO_2_ significantly increased expression of anti-inflammatory interleukin 10 (*IL10*) (*p* < 0.05) (Fig. 7b) after 4 h of treatment.

Expression of *INOS*, *TNFA*, *IL6* and IL1B was increased while *IL6* decreased after 24 h (*p* < 0.001) of exposition with LPS (Fig. 7c). No statistically significant differences were noted in the expression of *IL10, IL4* and *TGFB* after 24 h of LPS stimulation (Fig. 7d). In addition, HfO_2_ alone did not affect macrophages activation suggesting its biocompatibility. The obtained results indicate on anti-inflammatory properties of HfO_2_ .

## Discussion

In presented paper we described a deposition of a smooth amorphous layers of HfO_2_ using the ALD process and its characterization studies with XRD and AFM. It is well known that, the selection of the ALD parameters determines the surface structure. The temperature and the number of cycles are most crucial parameters affecting ALD [11]. The layers obtained in the low-temperature ALD process are amorphous, which in turn ensures perfect adhesion to the substrate [24]. On the other hand, appropriate selection of ALD cycles determines the thickness of the layer. As we have shown in our previous research, hafnium (IV) oxide coatings tend to crystallize during deposition of the thicker layers and at increased process temperature [24]. Considering the specific growth rate (1.9Å/cycle) for the selected process at the temperature of 90 °C, here we performed the 100 nm deposition process, which was confirmed by measuring film thickness using reflectometry. The characteristic feature of ALD is thickness adjustment of the films by determining the number of full ALD cycles in relation to the growth rate under given thermodynamic conditions [9].

Strong adhesion of the amorphous layer with the substrate will guarantee its strength in conditions of mechanical load to which hard tissue implants are subjected. Among the high material requirements for implant coatings a lack of cracks, both on the surface and on the interface of the materials, should be considered. Damage on the interfaces weakens the mechanical properties of the coating and, propagating towards the surface, can cause separation of micro fragments of the layer, which will lead to destruction of the coating during use of the implant.

XPS analysis of the chemical composition showed an excess of oxygen on the surface. About 25% of the oxygen was associated with surface pollution, clearly more than stoichiometric value for HfO_2_. This indicate on hydrophilic nature of the substrate, which significantly improves the biological properties of the surface [25]. Ultimately, the ratio of oxygen to hafnium is 1.86, which is close to the ideal stoichiometry. The lower ratio of O to Hf during sputtering results from the fact that 2 keV Ar ions preferentially remove O from the surface. Nevertheless, XPS depth profiling confirmed the homogeneity of the formed film.

Although the HfO_2_ layer is a widely studied material by many research groups [26, 27], its application in regenerative medicine is a new approach. In our previous research we have showed that HfO_2_ films obtained by ALD technology are characterized by high cyto-compatibility and antimicrobial properties [28, 29],. In the present study, we investigated whether HfO_2_ layers are able to modulate metabolic activity of both osteoblasts and osteoclasts and thus may be utilized in the fabrication of implants used in the fixation of osteoporotic bone fractures. Since now, limited number of studies regarding biological properties of hafnium oxide have been performed and none of them related to bone remodeling. A study performed by Fohlerova and Mozalev revealed that hafnium-oxide thin films on substrates via self-organized electrochemical anodization were characterized by good cytocompatibility (tested with MG-63 osteoblast-like cells) and anti-microbial properties (tested with Gram-negative *E. coli* bacteria) [30]. It was also shown that hafnium oxide nanoparticles can induce DNA damage and apoptosis in human colorectal cancer cells [31]. Cytotoxicity of hafnium oxide nanoparticles was also tested by Field et al. [32]. who revealed that these particles are relatively non-toxic in human HaCaT skin cells. Here we observed, that HfO_2_ promotes pre-osteoblasts, while inhibits pre-osteoclasts viability. What is more important, HfO_2_ protects pre-osteoblast against apoptosis through enhanced expression of BCL-2 transcript and at the same time induces pre-osteoclasts apoptosis. Obtained data delivers interesting scientific findings which can be utilized as a strategy in the course of osteoporotic bone regeneration via silencing osteoclasts overactivity [33]. Interestingly, we observed, that HfO_2_ protects pre-osteoblasts against apoptosis not only by activation particular osteogenesis related genes but also by induction of miR-17-5p and miR-7a-5p expression-crucial regulators of osteoblasts proliferation activity as well as inhibition of apoptosis [34, 35]. For that reason, we speculate that that the application of HfO_2_ on the surface of implantable metallic materials might become an effective anti-apoptotic strategy for osteoblast protection and at the same time enhance regenerative potential.

Restoring the balance between bone formation and bone resorption processes during OP is thought to be the most effective approach to prevent fractures and improve tissue homeostasis [36]. In the present study, we have found, that HfO_2_ promotes expression of runt-related transcription factor 2 (RUNX2), transforming growth factor β encoded by TGFB and osteopontin (OPN) in osteoblasts on the both mRNA as well as protein level. In numerous studies, RUNX2 has been shown to participate in osteoblast growth and differentiation by triggering the expression of subsequent biomarkers of mature osteoblasts [36–39]. Moreover, RUNX2 is up-regulated in early stages of osteoblast differentiation, then down-regulated in mature osteoblasts [40].

Obtained results indicated on beneficial effect of HfO_2_ in the context of the osteogenesis initiation. Moreover, we have demonstrated, that pre-osteoblasts exposed for HfO_2_ are characterized by enhanced expression of miR-21-5p and miR-16-5p- two critical transcripts involved in osteogenesis and bone mineralization. Interestingly, when compared to control cells, HfO_2_ did not promote osteocalcin expression, which mediates biomineralization during osteogenic maturation [41]. For that reason, we speculate that HfO_2_ might serve as an bioactive agent able to modulate and trigger osteoblastogenesis. On the other hand, we have shown that besides activation of osteogenesis, HfO_2_ inhibits osteoclastogenesis. We have shown, that HfO_2_ reduce the expression of key factors regulating osteoclasts differentiation- c.FOS, MMP9, PU.1, RANK and TRAP. Furthermore, It was also shown, that certain proinflammatory cytokines could induce osteoclastogenesis and can target osteoclasts via directly or via RANK/OPG axis [42]. Numerous studies indicated on systemic and local inflammation as an events negatively affecting bone remodeling supporting the theory regarding inflammation-related OP pathogenesis [43]. In the present study we have shown, that HfO_2_ exerts immunomodulatory effect, since it increased expression of anti-inflammatory *IL10* in LPS-treated macrophages propagated onto HfO_2_. Moreover, we noted significantly reduced expression of IL-6 which is common cytokine characteristic for osteoporosis [44]. Obtained results strongly suggests, that HfO_2_ exerts anti-inflammatory effect, which might become an important factor, when its application in future implantology is considered.

## Conclusions

Recently, there is an urgent need to develop a smart, bioactive materials for OP bone regeneration that would be able to modulate osteoblasts-osteoclasts axis in order to improve fracture healing. In the present study, using ADL technology we obtained homogeneous, amorphous layer of HfO_2_ which exerted anti-apoptotic and pro-osteogenic effects in vitro. We have shown, that HfO2 promotes osteogenic differentiation through the activation of early markers of osteogenesis while inhibits viability and maturation of osteoclasts. Furthermore, HfO2 was characterized by immunomodulatory effects which shed a promising light for its future application as an active layer for covering metallic materials.

## Methods

### Substrate preparation

The HfO_2_ layers were deposited on 10 mm x10 mm silicon plates for physicochemical evaluations and on glass cover slips with a diameter of 12 mm for biological evaluation. Superficial contaminants may affect the adherence of the ALD films to the substrate and negatively affect the homogeneity of the layer. Thus, we have prepared the substrates by three washing cycles before the deposition process. The first bath was carried out in isopropanol, the next two in deionized water. All of them were performed in the ultrasound cleaner with bath in 37 °C. Afterwards all substrates were dried in nitrogen of 5 N purity.

### ALD technology

A Savannah-100 Cambridge NanoTech ALD reactor was used to perform the deposition of HfO_2_ films. We selected Tetrakis(dimethylamido)hafnium (TDMA-Hf, Hf[(CH_3_)_2_N]_4_, CAS number 19782-68-4) as the metal precursor and deionized water as the oxygen precursor. Forming of HfO_2_ occurs through a double exchange reaction [45]:

$${\text{Hf}}\left[ {\left( {{\text{CH}}_{ 3} } \right)_{ 2} {\text{N}}} \right]_{ 4} + {\text{ 2H}}_{ 2} {\text{O }}{-\!\!-} > {\text{HfO}}_{ 2} + {\text{ 4HN}}\left( {{\text{CH}}_{ 3} } \right)_{ 2} .$$

Precursors cycles were introduced sequentially into the growth chamber separated by purge phase of a growth chamber with nitrogen (6 N). The pulse times were equal 0.2 s and 0.04 s for hafnium precursor and for oxidant precursor respectively, with purge times equal 8 s after hafnium precursor doses and 22 s after oxygen precursor doses. The growth process of HfO_2_ consisted of 519 ALD cycles. The creation of films was carried out in the temperature 90 °C and vacuum below 66 Pa. At such process parameters the deposition rate was equal to 1.9 Å per one ALD cycle.

### Tests equipment for physicochemical analysis

To determine the thickness of the layer a NanoCalc 2000-UV/VIS (Micropack GmbH) system with specific software was used. The layer thickness was measured on a silicon substrate. The reflection spectrum from the silicon substrate without the layer was used as a reference measurement.

The crystallinity of the coatings was checked by XRD investigations. A Panalytical X’Pert Pro MRD diffractometer was used, equipped with an X-ray tube generating radiation at a wavelength of 1.54056 Å, a hybrid two-bounce Ge (220) monochromator, and a Pixcel detector.

A Scienta R4000 hemispherical analyzer (with pass energy 200 eV) and non-monochromatic Al Kα emission line with excitation energy equal to 1486.6 eV, was used for XPS measurements. At those experimental setting, the measured full width at half maximum of the Au 4f7/2 line was 1.1 eV. The carbon (C) 1 s line was bonded with the energy 285 eV and used to calibrate the energy scale. The neutralization gun was applied to avoid meaningful sample charging. A notable amount of carbon was found in as received sample. The depth profiling with 2 kV Ar ion gun was performed to test if the carbon was immanent at the surface only and to check the film homogeneity. The spectra were analysed using CASA program (Casa Software Ltd version 2.3.17).

Surface morphology was examined using the AFM Dimension Icon from Bruker. The equipment was operated in the PeakForce Tapping mode using silicon nitride SCANASYST-AIR probe with tip radius of 2 nm. The applied mode of operation allows imaging of the surface based directly on interaction forces between the probe and the sample. In addition, the ScanAsyst algorithm was used for the measurements. It allows continuous monitoring the image quality and automatic optimization of the selected parameters (ScanAsyst Auto Gain and ScanAsyst Auto Setpoint) according to sample condition during scanning. Measurements were performed in air under ambient conditions with a scan rate of 0.70 Hz. Each area was scanned with 512 × 512 pixels resolution. AFM data analysis was made with NanoScope Analysis software.

### In vitro cell culture

Murine MC3T3-E1 pre-osteoblasts were cultured in Minimum Essential Media Alpha without ascorbic acid (MEM-α, Gibco™ Thermo Fisher Scientific, MA, USA) supplemented with 10% feta bovine serum (FBS, Sigma Aldrich, Munich, Germany). The murine osteoclast precursor cell line 4B12 was kindly provided by Shigeru Amano from Department of Oral Biology and Tissue Engineering, Meikai University School of Dentistry [46]. The 4B12 cells were propagated in complete growth medium consisting of α-MEM (Sigma Aldrich, Munich, Germany) supplemented with 30% of calvaria-derived stromal cell conditioned medium (CSCM) and 10% FBS. Murine macrophage cell line RAW 264.7 was cultured in complete growth medium consisted of Dulbecco’s Modified Eagle Medium (DMEM, Sigma Aldrich, Munich, Germany) with 500 mg/L glucose supplemented with 10% FBS. Cells were cultivated in the CO_2_ incubator at constant conditions (37 °C, 5% CO_2_, and 95% humidity). Medium was refreshed every 2–3 days. The cells were passaged when population reached 80% confluence using recombinant cell-dissociation enzyme StableCell Trypsin (Sigma Aldrich, Munich, Germany) or by gentle scrapping and pipetting (4B12).

### Cell proliferation assay

Cell proliferation rate was estimated using a resazurin-based assay kit (TOX8) (Sigma Aldrich, Munich, Germany) in accordance to manufacturer’s protocol [47]. For the assay MC3T3-E1 and 4B12 cells were seeded in 24-well plates on regular glass slides (control group) or on glass slides coated with HfO_2_ at a density of 2·10^4^ cell/well. The test was performed at 48, 72 and 144 h of experiment. Briefly, culture media was replaced with fresh media supplemented with 10% v/v resazurin dye, and incubation was carried out for 2 h at 37  °C in the CO_2_ cell culture incubator (Thermo Fisher Scientific, MA, USA). Then, supernatants were transferred to 96-well plate in the volume of 100 μl per well and measured spectrophotometrically at a wavelength of 600 nm and 690 nm reference length (Epoch, Biotek, Germany) Population doubling time (PDT) was calculated using an algorithm available on-line [48].

### Macrophage activation

RAW 264.7 cells were seeded in 24-well plate on regular glass slides (control) or on glass slides coated with HfO_2_ a density of 3·10^5^ cells/well. After 24 h lipopolysaccharide (LPS, 1 μg/mL, Sigma Aldrich, Munich, Germany) was added to culture media for another 4 and 24 h. Macrophage activation was analyzed using RT-qPCR technique and confocal microscopy.

### Confocal microscopy

Cellular distribution and expression of runt-related transcription factor 2 (RUNX2), osteoprotegerin (OPG), and osteopontin (OPN) in MC3T3-E1 cells were determined using immunofluorescence [49]. Cells were cultivated for 6 days on glass slides with and without HfO_2_ layer (control group). First, cells were fixed with 4% paraformaldehyde (PFA) for 30 min at room temperature, washed three times with Phosphate Buffered Saline (PBS, Sigma Aldrich, Munich, Germany), and then incubated with 0.2% Tween 20 (Sigma Aldrich, Munich, Germany) solution in PBS for another 15 min. Then, samples were blocked with 5% goat serum (Sigma Aldrich, Munich, Germany) for 1 h and incubated with primary antibodies at 4  °C overnight. The following antibodies were used: OPN (dilution 1:1000 in PBS, ab8448, Abcam, Cambridge, UK), OPG (dilution 1:50 in PBS, sc-390518, Santa Cruz Biotechnology, Dallas, TX, USA), RUNX2 (dilution 1:50 in PBS, sc-390351, Santa Cruz Biotechnology, Dallas, TX, USA). After the primary antibody incubation, cells were rinsed three times with PBS and incubated with secondary antibodies conjugated with Alexa Fluor 488 (room temperature, 1 h). Finally, cells were fixed on slides and nuclei were counterstained using mounting medium with DAPI (4’,6-diamidino-2-phenylindole) (ProLong™ Diamond Antifade Mountant with DAPI, Thermo Fisher Scientific, Warsaw, Poland). Cells were observed and imaged using confocal microscope (LEICA TSC SPE). Photos were analyzed with Image J software.

Changes in RAW 264.7 morphology after LPS-induced activation were estimated using F-actin staining. Prior staining, cells were fixed with 4% PFA after 4 and 24 h incubation with LPS and permeabilized using 0.2% Tween 20 as described above. Phalloidin Atto 590 was diluted in PBS (1:800, Sigma Aldrich, Munich, Germany) and added to cells for 40 min. After three washing steps cells were mounted using ProLong™ Diamond Antifade Mountant with DAPI, observed and imaged using confocal microscope (LEICA TSC SPE).

### Reverse transcription quantitative polymerase chain reaction (RT-qPCR)

Total RNA was isolated from MC3T3-E1 and 4B12 after 6 days of culture in normal condition or in the presence of HfO_2_, whereas total RNA extraction from RAW 264.7 was performed after 4 and 24 h of cultivation in normal condition or in the presence of HfO2 and/or LPS. RNA was isolated using phenol–chloroform method, as previously described by Chomczynski and Sacchi [50]. RNA quality and quantity were estimated spectrophotometrically at 260 and 280 nm wavelengths (Epoch, Biotek, Germany). thic.

Nawrocka et al. [51] for gene expression analysis, total RNA (150 ng per one reaction) was treated with DNase I using PrecisionDNAse kit (BLIRT S.A, Gdansk, Poland) followed by cDNA synthesis using RevertAidFirst Strand cDNA Synthesis Kit (Thermo Fisher Scientific, MA, USA). cDNA was subjected to real-time PCR with 5 µl of SensiFast SYBR & Fluorescein Kit (Bioline, UK), 1 µl of cDNA and 500 nM of each specific primer (see Table 1) in the total volume of 10 μl. All qPCR reactions were performed using CFX Connect™ Real-Time PCR Detection System (Bio-Rad, Hercules, CA, USA). Obtained data was normalized to the mean of GAPDH used as the housekeeping gene. The average fold change in the gene expression was analyzed using the 2^−ΔΔCT^ method described by Livak and Schmittgen [52]. Furthermore, the expression ratio of *BCl2/BAX* was determined by dividing the ΔΔCT of those genes.

**Table 1:** Sequence of primers used for mRNA expression analysis using RT-qPCR method

Gene abbreviation	Gene full name	Sequence of primer (5′ → 3′)	Amplicon lenght	Accession No.
*CASP9*	Caspase-9	F: CCGGTGGACATTGGTTCTGG	278	NM_001355176.1
		R: GCCATCTCCATCAAAGCCGT		
*P53*	p53 tumor suppressor	F: AGTCACAGCACATGACGGAGG	287	XM_030245924.1
		R: GGAGTCTTCCAGTGTGATGATGG		
*BCl2*	B-cell lymphoma 2	F: GGATCCAGGATAACGGAGGC	141	NM_009741.5
		R: ATGCACCCAGAGTGATGCAG		
*BAX*	Bcl-2-like protein 4	F: AGGACGCATCCACCAAGAAGC	251	XM_011250780.3
		R: GGTTCTGATCAGCTCGGGCA		
*TGFB*	Transforming growth factor beta 1	F: GGAGAGCCCTGGATACCAAC	94	NM_011577.2
		R: CAACCCAGGTCCTTCCTAAA		
*RUNX2*	Runt-related transcription factor 2	F: TCCGAAATGCCTCTGCTGTT	130	NM_001271630.1
		R: GCCACTTGGGGAGGATTTGT		
*OPN*	Osteopontin	F: AGACCATGCAGAGAGCGAG	340	NM_001204203.1
		R: GCCCTTTCCGTTGTTGTCCT		
*OCN*	Osteocalcin	F: GGTGCAGACCTAGCAGACACCA	100	NM_001032298.3
		R: CGCTGGGCTTGGCATCTGTAA		
*c.FOS*	c-fos proto-oncogene	F: CCAGTCAAGAGCATCAGCAA	248	NM_010234.3
		R: TAAGTAGTGCAGCCCGGAGT		
*MMP9*	Matrix metallopeptidase 9	F: TTGCCCCTACTGGAAGGTATTAT	172	XM_006498861.3
		R: GAGAATCTCTGAGCAATCCTTGA		
*PU.1*	Transcription factor PU.1	F: GAGAAGCTGATGGCTTGGAG	175	XM_017316733.2
		R: TTGTGCTTGGACGAGAACTG		
*RANK*	Receptor activator of nuclear factor κ B	F: TTAAGCCAGTGCTTCACGGG	473	NM_009399.3
		R: ACGTAGACCACGATGATGTCGC		
*TRAP*	Tartrate-resistant acid phosphatase	F: GTCTCTGGGGGACAATTTCTACT	241	XM_006509945.3
		R: GTTTGTACGTGGAATTTTGAAGC		
*CR1A*	Calcitonin receptor isoform 1a	F: TGCGGCGGGATCCTATAA	238	NM_001355192.1
		R: AGCCAGCAGTTGTCGTTGTA		
*INOS*	Nitric oxide synthase	F: GACAAGCTGCATGTGACATC	325	NM_001313922.1
		R: GCTGGTAGGTTCCTGTTGTT		
*TNFA*	Tumor necrosis factor α	F: ACAGAAAGCATGATCCGCGA	295	NM_013693.3
		R: CTTGGTGGTTTGCTACGACG		
*IL6*	Interleukin 6	F:GAGGATACCACTCCCAACAGACC	141	NM_001314054.1
		R:AAGTGCATCATCGTTGTTCATACA		
*IL1B*	Interleukin 1 beta	F: TGCCACCTTTTGACAGTGATG	138	NM_008361.4
		R: TGATGTGCTGCTGCGAGATT		
*L10*	Interleukin 10	F: ATTTGAATTCCCTGGGTGAGAAG	75	NM_010548.2
		R: CACAGGGGAGAAATCGATGACA		
*IL4*	Interleukin 4	F: GAATGTACCAGGAGCCATAT	385	NM_021283.2
		R: CTCAGTACTACGAGTAATCCA		
*GAPDH*	Glyceraldehyde 3-phosphate dehydrogenase	F: TGCACCACCAACTGCTTAG	177	XM_017321385.2
		R: GGATGCAGGGATGATGTTC		

For miRNA expression analysis, 500 ng of total cellular RNA was served for digestion of gDNA, as described above. RNA was polyadenylated and reverse transcribed using the Mir-X miRNA First-Strand Synthesis Kit (Takara Bio, Japan) according to the manufacturer’s protocol. Each qPCR reaction was performed in a total reaction mixture volume of 25 μl. Expression data were normalized using the 2^−ΔΔCT^ method after normalization to the U6 snRNA used as a housekeeping gene. Sequence of the primers are listed in the Table 2.

**Table 2:** Sequence of primers used for miRNA expression analysis using qPCR method

miRNA	Sequence of primer (5′ → 3′)	Accession No.
miR-7a-5p	TGGAAGACTAGTGATTTTGTTGT	MIMAT0000677
miR-17-5p	CAAAGTGCTTACAGTGCAGGTAG	MIMAT0000070
miR-21-5p	TAGCTTATCAGACTGATGTTGA	MIMAT0000530
miR-16-5p	TAGCAGCACGTAAATATTGGCG	MIMAT0000069

### Statistical analysis

All experiments included at least three technical repetitions. Comparison between two groups were analyzed using two-tailed Student’s *t* test. Differences among more than two groups were analyzed using two-way ANOVA analysis of variance followed by Bonferroni post hoc testes. Values of *p* < 0.05 were considered statistically significant. All data are means ±  SD.

## Data Availability

The datasets used and/or analysed during the current study are available from the corresponding author on reasonable request.

## References

[1] Miller RR, Roubenoff R (2019). emerging interventions for elderly patients-the promise of regenerative medicine. Clin Pharmacol Ther.

[2] Akkawi I, Zmerly H (2018). Osteoporosis: Current Concepts. Joints..

[3] Hernlund E, Svedbom A, Ivergård M, Compston J, Cooper C, Stenmark J (2013). Osteoporosis in the European Union: medical management, epidemiology and economic burden. A report prepared in collaboration with the International Osteoporosis Foundation (IOF) and the European Federation of Pharmaceutical Industry Associations (EFPIA). Arch Osteoporos..

[4] Prakasam M, Locs J, Salma-Ancane K, Loca D, Largeteau A, Berzina-Cimdina L. Biodegradable materials and metallic implants—a review. J Funct Biomater. 2017; (cited 2020 May 3); 8. https://www.ncbi.nlm.nih.gov/pmc/articles/PMC5748551/. 10.3390/jfb8040044 PMC5748551 28954399

[5] Prasad K, Bazaka O, Chua M, Rochford M, Fedrick L, Spoor J, et al. Metallic biomaterials: current challenges and opportunities. Materials (Basel). 2017; (cited 2020 May 3); 10. https://www.ncbi.nlm.nih.gov/pmc/articles/PMC5578250/. 10.3390/ma10080884 PMC5578250 28773240

[6] Eliaz N. Corrosion of Metallic biomaterials: a review. Materials (Basel). 2019; (cited 2020 May 3). 12. https://www.ncbi.nlm.nih.gov/pmc/articles/PMC6384782/. 10.3390/ma12030407 PMC6384782 30696087

[7] Oviroh PO, Akbarzadeh R, Pan D, Coetzee RAM, Jen T-C (2019). New development of atomic layer deposition: processes, methods and applications. Sci Technol Adv Mater.

[8] Im H, Wittenberg NJ, Lindquist NC, Oh S-H (2012). Atomic layer deposition (ALD): a versatile technique for plasmonics and nanobiotechnology. J Mater Res.

[9] Suntola T (1989). Atomic Layer Epitaxy.

[10] Knez M, Nielsch K, Niinistö L. Synthesis and surface engineering of complex nanostructures by atomic layer deposition. Advanced Materials. 2007. p. 3425–38.

[11] Miikkulainen V, Leskelä M, Ritala M, Puurunen RL (2013). Crystallinity of inorganic films grown by atomic layer deposition: overview and general trends. J Appl Phys..

[12] Knez M (2012). Application of ALD to biomaterials and biocompatible coatings. Atomic layer deposition of nanostructured materials.

[13] Graniel O, Weber M, Balme S, Miele P, Bechelany M (2018). Biosensors and bioelectronics atomic layer deposition for biosensing applications. Biosens Bioelectronic..

[14] Seweryn A, Pielok A, Lawniczak-Jablonska K, Pietruszka R, Marcinkowska K, Sikora M (2020). Zirconium oxide thin films obtained by atomic layer deposition technology abolish the anti-osteogenic effect resulting from miR-21 inhibition in the pre-osteoblastic MC3T3 cell line. Int J Nanomed.

[15] Marycz K, Sobierajska P, Roecken M, Kornicka-Garbowska K, Kępska M, Idczak R (2020). Iron oxides nanoparticles (IOs) exposed to magnetic field promote expression of osteogenic markers in osteoblasts through integrin alpha-3 (INTa-3) activation, inhibits osteoclasts activity and exerts anti-inflammatory action. J Nanobiotechnol..

[16] Marycz K, Grzesiak J, Wrzeszcz K, Golonka P. Adipose stem cell combined with plasma-based implant bone tissue differentiation in vitro and in a horse with a phalanx digitalis distalis fracture: a case report. Veterinarni Medicina (Czech Republic) [Internet]. 2012

[17] Rudenja S, Minko A, Buchanan DA (2010). Low-temperature deposition of stoichiometric HfO 2 on silicon: analysis and quantification of the HfO 2/Si interface from electrical and XPS measurements. Appl Surf Sci..

[18] Crist BV (2005). Handbooks of monochromatic XPS Spectra.

[19] Sokolov AA, Filatova EO, Afanas’ Ev VV, Taracheva EY, Brzhezinskaya MM, Ovchinnikov AA (2008). Interface analysis of HfO2 films on (1 0 0) Si using x-ray photoelectron spectroscopy. J Phys D Appli Phys..

[20] Wang J-X, Jia X-J, Liu Y, Dong J-H, Ren X-M, Xu O (2020). Silencing of miR-17-5p suppresses cell proliferation and promotes cell apoptosis by directly targeting PIK3R1 in laryngeal squamous cell carcinoma. Cancer Cell Int.

[21] Li S, Lv X, Zhai K, Xu R, Zhang Y, Zhao S (2016). MicroRNA-7 inhibits neuronal apoptosis in a cellular Parkinson’s disease model by targeting Bax and Sirt2. Am J Transl Res..

[22] Smieszek A, Marcinkowska K, Pielok A, Sikora M, Valihrach L, Marycz K (2020). The Role of miR-21 in osteoblasts-osteoclasts coupling in vitro. Cells.

[23] Mencía Castaño I, Curtin CM, Duffy GP, O’Brien FJ (2019). Harnessing an inhibitory role of miR-16 in osteogenesis by human mesenchymal stem cells for advanced scaffold-based bone tissue engineering. Tissue Eng Part A.

[24] Gieraltowska S, Wachnicki L, Witkowski BS, Mroczynski R, Dluzewski P, Godlewski M (2015). Characterization of dielectric layers grown at low temperature by atomic layer deposition. Thin Solid Films..

[25] Bang SM, Moon HJ, Kwon YD, Yoo JY, Pae A, Kwon IK (2014). Osteoblastic and osteoclastic differentiation on SLA and hydrophilic modified SLA titanium surfaces. Clin Oral Implant Res.

[26] Zhang X-Y, Hsu C-H, Lien S-Y, Wu W-Y, Ou S-L, Chen S-Y, et al. Temperature-dependent HfO2/Si interface structural evolution and its mechanism. Nanoscale Res Lett. 2019; (cited 2020 May 3). 14. https://www.ncbi.nlm.nih.gov/pmc/articles/PMC6405792/. 10.1186/s11671-019-2915-0 PMC6405792 30847661

[27] Xu J, Wen M, Zhao X, Liu L, Song X, Lai P-T (2018). Effects of HfO2 encapsulation on electrical performances of few-layered MoS2 transistor with ALD HfO2 as back-gate dielectric. Nanotechnology..

[28] Liu L, Bhatia R, Webster TJ (2017). Atomic layer deposition of nano-TiO 2 thin films with enhanced biocompatibility and antimicrobial activity for orthopedic implants. Int J Nanomed.

[29] Godlewski M, Gierałtowska S, Wachnicki Ł, Pietuszka R, Witkowski BS, Słońska A (2017). High-k oxides by atomic layer deposition—applications in biology and medicine. J Vacuum Sci Technol A.

[30] Fohlerova Z, Mozalev A (2019). Anodic formation and biomedical properties of hafnium-oxide nanofilms. J Mater Chem B R Soc Chem.

[31] Marill J, Mohamed Anesary N, Paris S (2019). DNA damage enhancement by radiotherapy-activated hafnium oxide nanoparticles improves cGAS-STING pathway activation in human colorectal cancer cells. Radiother Oncol.

[32] Field JA, Luna-Velasco A, Boitano SA, Shadman F, Ratner BD, Barnes C (2011). Cytotoxicity and physicochemical properties of hafnium oxide nanoparticles. Chemosphere.

[33] Charles JF, Aliprantis AO (2014). Osteoclasts: more than ‘bone eaters’. Trends Mol Med..

[34] Wang X, Li Z, Bai J, Song W, Zhang F (2019). miR-17-5p regulates the proliferation and apoptosis of human trabecular meshwork cells by targeting phosphatase and tensin homolog. Molecular Medicine Reports. Spandidos Publications.

[35] Jia J, Feng X, Xu W, Yang S, Zhang Q, Liu X (2014). MiR-17-5p modulates osteoblastic differentiation and cell proliferation by targeting SMAD7 in non-traumatic osteonecrosis. Exp Mol Med.

[36] Guido G, Scaglione M, Fabbri L, Ceglia MJ (2009). The “osteoporosis disease”. Clin Cases Miner Bone Metab..

[37] Ling M, Huang P, Islam S, Heruth DP, Li X, Zhang LQ (2017). Epigenetic regulation of Runx2 transcription and osteoblast differentiation by nicotinamide phosphoribosyltransferase. Cell Biosci.

[38] Komori T (2010). Regulation of osteoblast differentiation by Runx2. Adv Exp Med Biol.

[39] Kawane T, Qin X, Jiang Q, Miyazaki T, Komori H, Yoshida CA (2018). Runx2 is required for the proliferation of osteoblast progenitors and induces proliferation by regulating Fgfr2 and Fgfr3. Sci Rep.

[40] Komori T. Regulation of Proliferation, Differentiation and Functions of Osteoblasts by Runx2. Int J Mol Sci. 2019 (cited 2020 Apr 1);20. https://www.ncbi.nlm.nih.gov/pmc/articles/PMC6480215/. 10.3390/ijms20071694 PMC6480215 30987410

[41] Moser SC, van der Eerden BCJ. Osteocalcin—a versatile bone-derived hormone. Front Endocrinol (Lausanne). 2019 (cited 2020 May 3);9. https://www.ncbi.nlm.nih.gov/pmc/articles/PMC6335246/. 10.3389/fendo.2018.00794 PMC6335246 30687236

[42] Zupan J, Jeras M, Marc J (2013). Osteoimmunology and the influence of pro-inflammatory cytokines on osteoclasts. Biochem Med (Zagreb)..

[43] Ginaldi L, Di Benedetto MC, De Martinis M (2005). Osteoporosis, inflammation and ageing. Immun Ageing..

[44] Edwards CJ, Williams E (2010). The role of interleukin-6 in rheumatoid arthritis-associated osteoporosis. Osteoporos Int.

[45] Leskelä M, Ritala M (2002). Atomic layer deposition (ALD): from precursors to thin film structures. Thin Solid Films.

[46] Amano S, Chang Y-T, Fukui Y (2015). ERK5 activation is essential for osteoclast differentiation. PLoS ONE.

[47] Starosta R, Brzuszkiewicz A, Bykowska A, Komarnicka UK, Bażanów B, Florek M (2013). A novel copper(I) complex, [CuI(2,2′-biquinoline)P(CH2N(CH2CH2)2O)3] – Synthesis, characterisation and comparative studies on biological activity. Polyhedron..

[48] Doubling Time—online computing with 2 points. (cited 2018 Oct 3). http://www.doubling-time.com/compute.php.

[49] Marycz K, Kornicka K, Grzesiak J, Śmieszek A, Szłapka J. Macroautophagy and Selective Mitophagy Ameliorate Chondrogenic Differentiation Potential in Adipose Stem Cells of Equine Metabolic Syndrome: New Findings in the Field of Progenitor Cells Differentiation [Internet]. Oxidative Medicine and Cellular Longevity. 2016 [cited 2019 Jun 27] 10.1155/2016/3718468 PMC5178365 28053691

[50] Chomczynski P, Sacchi N (1987). Single-step method of RNA isolation by acid guanidinium thiocyanate-phenol-chloroform extraction. Anal Biochem.

[51] Nawrocka D, Kornicka K, Śmieszek A, Marycz K. Spirulina platensis Improves Mitochondrial Function Impaired by Elevated Oxidative Stress in Adipose-Derived Mesenchymal Stromal Cells (ASCs) and Intestinal Epithelial Cells (IECs), and Enhances Insulin Sensitivity in Equine Metabolic Syndrome (EMS) Horses. Mar Drugs [Internet]. 2017 10.3390/md15080237 PMC5577592 28771165

[52] Livak KJ, Schmittgen TD (2001). Analysis of relative gene expression data using real-time quantitative PCR and the 2(-Delta Delta C(T)) Method. Methods.

